# Coping strategies used by undergraduate first-year nursing students during transition from basic to higher education: a qualitative study

**DOI:** 10.1186/s12912-024-01938-5

**Published:** 2024-04-24

**Authors:** Gopolang Gause, Leepile Alfred Sehularo, Molekodi Jacob Matsipane

**Affiliations:** NuMIQ Research Focus Area, Faculty of Health Sciences, North-West University, Potchefstroom, South Africa

**Keywords:** Basic education, Coping, Coping strategies, Higher education, Undergraduate, Undergraduate first-year nursing students, Transition

## Abstract

**Background:**

The undergraduate first year of a nursing program is regarded as a difficult and challenging part of the nursing course, due to the variation experienced in the transition from basic to higher education compared to other first-year courses. This causes stress, which could contribute to students’ lack of coping with the transition to the university. These challenges call for coping strategies to ensure resilience among this cohort of undergraduate nursing students.

**Methods:**

An exploratory descriptive qualitative research design was adopted to assess the coping mechanisms used by first-year nursing students during transition from basic to higher education. Data was gathered through four online focus group discussions which were conducted using the Microsoft Teams app, with participants purposefully selected from the two campuses where the study was conducted. The focus group discussions were recorded and transcribed verbatim for analysis. Data was analysed by the researcher and the co-coder using qualitative content data analysis.

**Findings:**

Three categories emerged from the data: experiences of transition from basic to higher education, coping with transition from basic to higher education, and recommendations for coping with transition from basic to higher education. Participants expressed that they use the following six coping strategies during the transition from basic to higher education: adaptive coping, appraisal-focused coping, emotion-focused coping, problem-focused coping, social coping, and seeking help from mentors.

**Conclusions:**

Transition from basic to higher education is challenging for undergraduate first-year nursing students. The study suggests that there is a need to standardize and contextualize the support measures for undergraduate first-year nursing students during their transition from basic to higher education in order to enhance their ability to cope.

## Background

Undergraduate first-year nursing students often find the adjustment to the university environment difficult and challenging due to the demands of the nursing program [1, 2]. This is because nursing programs are challenging and competitive in nature, as they encompass the need to satisfy two mutually important components of nursing education – academic and clinical requirements – prior to completion [1]. This implies that in addition to the need to satisfy the theoretical requirements, undergraduate first- year nursing students are also expected to report to clinical placement areas as early as their first semester [3]. For some, these demands cause a great deal of stress and a negative experience of the nursing program, which contributes to lack of coping with the transition to the university experienced by this cohort of undergraduate nursing students [2, 3].

On the clinical side of the nursing program, literature shows that undergraduate first- year nursing students face challenges that require coping mechanisms [4, 5]. Some challenges reported by undergraduate nursing students at clinical facilities include, among others, theory and practice discrepancies, inability to master the clinical skills taught, and discrepancies between real-life clinical requirements and inducted procedures [4]. In addition, a study conducted by Lavoie-Tremblay et al. [5] highlighted fear of harming the patient, demonstration of clinical skills under close supervision, poor knowledge about patients’ medical condition, and the need to care for a dying patient as additional stressors among the undergraduate first-year nursing students in clinical facilities. These challenges contribute to poor academic resilience among the cohort of students referred to in this study.

Similarly to the clinical challenges, there is evidence of challenges emanating from the theory side of the Bachelor of Nursing program [2, 6]. One challenge that is relatively obvious is the need to adapt to the teaching modalities used in higher education, which are totally different from those used during basic schooling [2, 6]. Furthermore, issues related to the curriculum, that is rich in content which translates to intensive lecture hours, long study hours to grasp the content, and daily schedules swamped with a variety of lectures covering different aspects of the curriculum, are challenging for undergraduate first-year nursing students [2, 5]. As a result, a significant number of undergraduate nursing students find it difficult to keep up with the whole process of transitioning into the university while also safeguarding the need to form and sustain other relations. This highlights the need for undergraduate first-year nursing students to be provided with much-needed support during their transition from basic to higher education [1].

Several authors have explored how undergraduate nursing students cope with their first year of the Bachelor of Nursing program [3, 4, 7]. In a study conducted by Alshahrani et al. [3] “venting out frustrations” to peers was earmarked as one of the coping strategies used to deal with academic stressors amid the transitioning period. Some authors suggest learning through others, especially at higher levels of training, as a key strategy to cope with transitioning to university for first year students [7, 8]. These studies include an emphasis on management of expectations and provision of support [7]. Although some undergraduate nursing students would resort to psychoactive substances to cope with academic stressors, a study conducted by Bodys-Cupak et al. [4] elucidated that the use of such substances is less common. Instead, the majority of the students use active coping mechanisms such as seeking support, while a significant number would go to the extent of ceasing the activities which they are not coping with [4].

Although there is a fair amount of literature on this phenomenon, as depicted above, there is a gap regarding the applicability of these recommendations for the current population of undergraduate first-year nursing students, whose high schooling took place entirely during the COVID-19 era. This suggested the need to conduct this study in the era of the denouement of COVID-19 and advancement of technology in higher education institutions as influenced by online teaching and learning as necessitated by the COVID-19 pandemic.

It is against this background that the researcher saw it worthwhile to conduct this study, which aimed to explore and describe the coping strategies used by undergraduate first-year nursing students during their transition from basic to higher education at a South African university.

## Methods

The methods that underpinned this study are discussed in this section.

### Research design

An exploratory descriptive qualitative [9] research design was adopted to achieve the aim of this study. Such a research design is best suited for studies in healthcare practice and education, including this one, as it allows for exploration and description of phenomena on aspects of nursing education and practice, including related policies [9].

### Study setting

The study was conducted at a university in South Africa, which has three campuses spread across two provinces. The third campus of this university was not yet accredited to offer the undergraduate nursing qualification at the time of conducting this study. The two campuses where the study was conducted offer an aligned undergraduate curriculum. Although the university strives to offer the same higher education experience to its students across its campuses, the two campuses vary in terms of geographic urbanization: one campus is located in a semi-urban area, while the other is in a predominantly rural area.

### Participants

The participants were undergraduate first-year nursing students from the two campuses of a university in South Africa. In the context of this study, undergraduate first-year nursing students are those who hold a Grade 12 certificate (university entry exam) and are in their first year of a four-year Bachelor of Nursing program. The researcher’s interest was piqued by literature that has shown that poor resilience is predominant among undergraduate first-year nursing students, especially those arriving at university straight from high school [7]. A non-probability purposive sampling technique was used to select potential participants and form four focus group discussion (FGD) groups from a population of 156 undergraduate first-year nursing students across the two campuses. The process of sampling was conducted with the assistance of a mediator to ensure that potential participants were thoughtfully selected. The participants included males and females aged between 18 and 21 years, the inclusion criterion being bona fide undergraduate first-year nursing students. First-year students in postgraduate qualifications, undergraduate first-year nursing students who are repeating first year and undergraduate nursing students at year levels 2, 3, and 4 were excluded.

### Data collection

Data was collected in August 2023 through online FGDs which were conducted using the Microsoft Teams app guided by an unstructured FGD schedule. The literature recommends that an FGD should comprise between five and twelve participants [10–13]. Two FGDs were held for each campus, comprising ten and seven participants for campus A, and seven and eight participants for campus B. The choice of FGD as a method was due to its capability of producing data rich in detail through widening the range of responses and activation of forgotten ideas [12]. Data was gathered during students’ free time, mostly after classes when they were mostly in their rooms and using institutional WiFi. For participants who did not stay at either a campus residence or accredited residences with free WiFi, a 1GB data bundle was provided so that they could connect and participate in the online FGD. Participants were requested to keep the cameras of their devices on during data collection, to ensure that all nonverbal cues were captured as field notes. The type of data that were collected were the demographic information of the participants and that relating to their coping mechanisms regarding transition from basic to higher education. In exploratory descriptive research, collecting quantitative materials such as the demographics of participants helps to support the results [9]. However, no identifying information such as names, identity numbers or student numbers were collected. Instead, participants were referred to by numbers, as participant 1, 2, 3 and so forth. Although the FGDs were primarily conducted in English, participants were awarded an opportunity to clarify in their vernacular since English was not their primary language. The FGDs were recorded using the recording option on Microsoft Teams and later transcribed verbatim. The vernacular transcriptions were translated by an expert in language, after which they were checked by a bilingual expert to ensure that they reflected the collected data. Each FGD lasted between 50 and 60 min.

### Trustworthiness

Trustworthiness was ensured through credibility, dependability, confirmability, and transferability [10, 11]. In addition, the authors used other measures of triangulation during data collection, where field notes from the video feed were collected to corroborate the findings. Furthermore, discrepant information that runs counter to the categories identified in this study was also described during the discussion. According to Creswell and Creswell [13], discussion of discrepant information adds to the credibility of the findings, because it is a common occurrence that life perspectives do not always coalesce. Finally, the findings in the form of categories and sub-categories were also confirmed with the participants, to ensure that the findings represented the data that was obtained from them, a method known as member checking.

### Data analysis

Data was analyzed following a conventional qualitative content data analysis method, encompassing a detailed analysis of narrative data to generate categories and patterns of these categories [11]. Conventional qualitative content analysis involves segmenting raw data into small units, followed by coding, and naming the units as per the content that they are representing, and finally grouping the generated codes to form categories and sub-categories [11]. To achieve this, data was analyzed independently by both the researcher and the co-coder throughout the data collection process. The second and third authors were available on standby in case there were conflicts regarding consensus on the categories between the researcher and co-coder. However, there were no such conflicts as the researcher and co-coder agreed on the categories and sub-categories. The following steps by Creswell and Creswell [13] in conjunction with Atlas.ti version 23 software were used for data analysis (see Table 1).

**Table 1:** Data analysis steps

Step description	Data analysis activity
Step 1: Organising and preparing the data for analysis	The researcher transcribed the recorded FGDs held using MS Teams, including typing the field notes observed from the video footage. Data was subsequently arranged according to the sources from which it was obtained
Step 2: Reading all the data	The researcher and the co-coder independently read through all the transcribed data in conjunction with the field notes to formulate an understanding of what the participants are saying.
Step 3: Coding all the data	Coding the data was done independently by the researcher and co-coder with the use of computer-aided qualitative data analysis software called Atlas.ti. The researcher loaded the transcribed data into Atlas.ti to initiate coding, which followed these steps: data structuring, generating codes, writing comments and memos, and finally generating outputs in the form of a code. Finally, the outputs generated by the researcher were compared with those from the co-coder to reach a consensus.
Step 4: Generating categories	Codes that were generated in step 3 and agreed upon by the researcher and co-coder were grouped together to identify similarities. After that general descriptions were made in relating codes to generate categories and sub-categories which were then discussed as the major findings of this study.
Step 5: Representing all the categories	In this step, the implications of the findings of this study were discussed in relation to the literature that is already in the public domain.

### Ethical considerations

The proposed study was defended and approved by the institutional scientific review committee. It was then sent to the institutional ethics review committee to ensure that it met the minimum standards of conducting research with human participants, and it was approved and issued with a unique ethics number (NWU-00053-23-A1). Upon approval by both the scientific and ethics committee, goodwill permission was obtained from the Research Data Gate Keeper Committee (NWU-GK-23-140) of the institution where the study was conducted. Finally, goodwill permission was sourced from the individual deputy directors of the campuses where data was collected. To ensure that participants’ right to autonomy was respected, recruitment was carried out by an independent research assistant because the researcher is a staff member at the selected university. This ensured that recruitment was fair and just, without coercion or undue influence. As a result, participation in the study was entirely voluntary. Participants were allowed at least 14 days to read through the informed consent and decide if they wanted to participate or not. It is no secret that FGDs cannot be 100% confidential, which was one of the key ethical issues [12]. However, to combat this, participants were requested to keep all matters being discussed in the FGD confidential. Furthermore, all research team members were requested to sign a standard institutional confidentiality agreement form to ensure confidentiality throughout.

## Findings

### Demographic data

Data was collected from four FGDs (two from each campus). The demographic characteristics of the participants are indicated in Table 2.

**Table 2:** Demographic characteristics of participants

Participants’ demographics	Focus group			
	1	2	3	4
Total number of participants	10	7	8	7
Gender Male Female	7	1	2	2
	3	6	6	5
Age (years) 18 19 20 21	4	4	6	6
	4	2	1	1
	1	-	1	-
	1	1	-	-
Race African White Mixed race Indian Other	10	7	5	6
	-	-	3	1
	-	-	-	-
	-	-	-	-
	-	-	-	-

The demographic data shows that the majority (*n* = 20) of the participants were females, with 12 male participants. This could be due to the fact that nursing remains a female- dominated profession to date. The age of the participants ranged between 18 and 21 years, with the mean of 18.56. Few (12.5%) of the participants where white, while the majority (87.5%) were black Africans. There were no participants from other racial backgrounds. All participants were bona fide undergraduate first-year nursing students.

### Categories and sub-categories

Three categories and sub-categories were identified from data analysis and are presented in Table 3.

**Table 3:** Categories and sub-categories

Categories	Sub-categories
Experiences of transition from basic to higher education	Transition to independence
	Transition to demanding schedule
	Adapting to theory demands
	Adapting to clinical demands
	Adapting to social systems
	Balancing clinical and theoretical demands
Coping with transition from basic to higher education	Adaptative coping
	Appraisal-focused coping
	Emotion-focused coping
	Problem-focused coping
	Social coping (peers)
	Seeking help from mentors
Recommendations for coping with transition from basic to higher education	Academic support
	Informational support (orientation)
	Psychological support
	Support from mentors
	External support (program planning and structure)
	Opportunities to improve communication
	Opportunities to release stress

## Category 1: experiences of transition from basic to higher education

The first category, experiences of transition from basic to higher education, had six sub-categories (Table 3), which are elaborated on below.

### Sub-category 1.1: transition to independence

Participants expressed that transition from basic to higher education implies transition to independence. This include shifting from traditional teaching modalities to more self-directed and student-centered learning. They explained that unlike in basic education, where their studies were primarily planned for by their parents, the transition to higher education saw them becoming fully responsible for organising their studies. According to the participants, the transition to independence is mainly because during higher education they mainly stay alone and do not have anyone to share responsibilities with, like their parents, siblings, or even helpers. Furthermore, although they felt that the transition from basic to higher education implies a transition to independence, participants alluded to the fact that it is a difficult and challenging process, especially in the first semester.

The following verbatim quotes from participants illustrate this:

*“I needed to get comfortable with the fact that I am living on my own and need to look after myself [showing quotation sign] if you want to call it that. And I think that thing that you need to go buy your own food, you need to make your food [laughs], you need to do your washing, you need to keep your apartment clean, things that we kind of [had] done by your parents or just ensured by them, it adds a lot of stress.”*

“But when you are in university, you need to focus more on the things like, I need my uniform by tomorrow, I’ve worn it yesterday, I need to wash it, but I have this and this and this classes today.”

### Sub-category 1.2: transition to demanding schedule

Participants expressed that they struggle with time management; lack of time management and increased workload during higher education make it difficult for them to manage their modules and weekly schedules. The participants’ explanation of the demanding schedule was centered around the theoretical demands of the undergraduate nursing degree, its clinical demands, and the need to maintain a social life. They explained that a weekly outline of their schedule would include classes which sometimes stretched up to two hours and 45 min, work-integrated learning placements and tests. They further explained that some classes would end at around 17h45 on Fridays, thus, negatively affecting their concentration span:

*“I think a lot of us isn’t coping, like I’m still struggling with my time management. And yeah, the workload.”*

*“it’s a little bit difficult for me to like to, to, to manage my modules because you find it maybe today or rather tomorrow, we have a test. Friday. Thursday. We have a test.”*

### Sub-category 1.3: adapting to theory demands

Participants elucidated that the teaching style during higher education seems too fast for them compared to that in basic education, which makes adapting to academic demands difficult. In addition to unfamiliar teaching styles, they expressed that one of the challenges that they grapple with is the rich content, which they are expected to grasp within a short space of time. They referred to the content at higher education as complex and always requiring an extra effort, or someone to guide them through the process of learning. According to the participants, learning in higher education is seen as a never-ending cycle which extends beyond the classroom premises:

*“The content from, okay, from high school is different from the content from varsity in a way that the varsity one is complex, and you cannot do it by yourself.”*

*“Let me just say, it was a bit difficult obviously, because of the learning and teaching. Adaptations differ and yeah, so it was like kind of hard for me to adapt.”*

### Sub-category 1.4: adapting to clinical demands

Participants described their experience of adapting to clinical demands during the transition from basic to higher education as similar to being thrown in the deep end. This is because adapting to clinical demands had to happen concurrently with trying to adapt to academic demands. They explained that they felt that they were not prepared enough psychologically to deal with cases such as psychiatric patients at outpatient departments, or caring for a dying patient. For some, this would include caring for a large number of sick patients, as this took an emotional toll on them. Furthermore, participants expressed that they experience being looked down on and ill-treated by other healthcare professionals, including students pursuing healthcare professions other than nursing. This makes their adaptation to clinical demands even more difficult. What also stood out was also the existence of stigma attached to male nursing students:

*“I felt like I was thrown in the deep end because I was a bit confused as to what to expect from the actual work that we are going to do, and the placements in hospital, and what’s going to be expected from us, while working in a hospital.”*

*“What we’ve been seeing, it is heavy stuff like seeing a patient pass away or dealing with a mentally ill patient for the first time. It’s not something a normal 18- or 19-year-old person faces.”*

### Sub-category 1.5: adapting to social systems

Participants expressed that transition from basic to higher education required them to adapt to unfamiliar social systems. They explained that they had challenges such as language barriers and culture shock at the institution of higher learning, which led them to be somewhat preoccupied. Some participants indicated that they had to learn to adapt to city life, since they were from primarily rural small villages with no exposure at all to city life. They further explained that, despite these unfamiliar social systems, they had to form social relations since they were stuck together and had to learn to survive together. Another major finding was that some of the participants were naturally introverts and had to learn how to socialize, as they were required to work in groups when completing some of the activities. Also, participants expressed that they were not sure of the boundaries of seeking help from their lecturers, compared to what they were used to during basic education:

*“I found it a little difficult since I graduated last year and came from a small town in the Western Cape. So, there were like really few people and then coming to Campus B, there was this this massive [laughs while talking] amount of people and cultures and different languages…”*

*“Now there is how many different people that you never met in your life before, its complete strangers, and now you have to not only study together the same thing but deal with these difficult situations of emotional heaviness together with your colleagues and its difficult.”*

### Sub-category 1.6: balancing clinical and theoretical demands

Participants expressed that they were overwhelmed with clinical and theoretical demands, and often find it difficult to balance the two. According to them, what exacerbates the lack of balance between clinical and theoretical demands is the staggering allocation. Another key element that they highlighted regarding balancing the clinical and theoretical demands was that they often return from clinical services late, at around 21h00, and they would be expected to be in class the following day, or vice versa. They state that this puts a strain on them and does not allow them time to fully prepare for the next day. As a result, participants felt that the pressure from the need to satisfy the clinical and theoretical demands does not allow them enough chance to self-study and revise the content taught in class or at the simulation room. One other key element that they highlighted was that sometimes they witness cases at the clinical sites which are unpleasant, but regardless of that they still need to adhere to the theoretical schedule, which could be a test the next day after such a traumatic event:

*“When she says balancing between going to placement and school also, academics. It’s like, Sir, we see a lot of traumatising things at the hospital. Also, at school the pressure is a lot. It’s like it’s kind of difficult to balance those two things, mental health …”*

*“… for example, this week we wrote, we were just writing today. Yesterday we were working. Tomorrow we are working, and we are writing on Friday. So, it is kind of difficult to catch up with the work, both theory and practical of course.”*

## Category 2: coping with transition from basic to higher education

The second category, coping with transition from basic to higher education, had six sub-categories (refer to Table 2).

### Sub-category 2.1: adaptive coping

Adaptive coping refers to the application of both cognitive and behavioural efforts towards the management of a stressful situation or event [14]. In this study participants used adaptive coping mechanisms to cope with the transition from basic to higher education. According to them the first point was to realize the need to change and accept it. This means that participants acknowledged that they were now in higher education and needed to learn to adapt to it. Those who had attended boarding school expressed that it was not really an adaptation process but an adjustment, since they had been semi-independent at school. A second adaptive coping strategy that they highlighted was the need to breech barriers like language by learning a few words of the language which they struggled with, for purposes of communication among peers and especially with patients. Participants expressed that they also had to draft their own timetables to improve on time management. They also explained that they use platforms like YouTube to boost their understanding of content after lectures and while studying:

“*I think also by learning small amounts of words from other languages, it helped, and then just going with the flow [laughs]. Ultimately it helps you in the end, you get comfortable.*”

*“Honestly, I think just for me personally, I think we just need to realize the change and just accommodate this change and adapt to the change, because I mean it gets different and difficult.*”

### Sub-category 2.2: appraisal-focused coping

Appraisal-focused coping is concerned with changing one’s mindset about a stressful situation or one they feel that they cannot cope with, in order to be able to cope with it [14]. The participants expressed that a change of mindset about higher education greatly assisted them to cope with the transition. They explained that they changed their mindset to focus on what other students are doing to be able to prosper in the Bachelor of Nursing degree, and tried to apply that in their lives too. Appraisal-focused coping was also applied, even in the clinical setting where participants expressed that they would sometimes put themselves in patients’ shoes to gain a better understanding of their situation. This assisted them to cope with cases, such as the management of psychiatric patients at clinics or in the outpatients’ department:

*“… I found a way to change my mindset, like focus more on knowing what people do in order to achieve and be successful in life, and try to apply that in in my own life, in my own daily living.*”

“… *making a mind change and realising that It’s not just you. There’s a bigger picture. But yeah, I don’t have a coping mechanism, a coping strategy for that. I just think that is a mindset change that you have to do.*”

### Sub-category 2.3: emotion-focused coping

An emotion-focused coping mechanism was identified as one of the strategies used by undergraduate first-year nursing students to cope with the transition from basic to higher education. An emotion-focused coping mechanism is defined as a process of the individual employing efforts aimed at reducing the stressors that they cannot cope with [14]. The participants expressed that while the main goal is to balance the clinical and theoretical demands, there is also a need to engage in recreational activities that are offered at the higher education institution, to reduce stress. In addition, participants stated that adequate sleep, doing house chores, and periodic exercise also assisted them to lower their stress level and be able to cope better with the transition. Socialising and listening to music were also aspects highlighted under emotion- focused coping strategy:

*“I can say try to balance your life, your social life and your academic life. For example, it’s not a matter of you must always be focused on your studies. Try to be part of the recreational activities…”*

*“And you also need to make time for res and to participate with that, and make sure that you have, and [that you are] actually enjoying it while you are studying.”*

### Sub-category 2.4: problem-focused coping

Problem-focused coping is a strategy where an individual directly faces a problem or a stressor that is responsible for poor coping [14]. In this study, the participants expressed that one of the potential problems in terms of lack of coping is the accessibility of entertainment. They explained that they had to reduce entertainment time, including time spent on social media, significantly. They indicated that the time that they had initially spent on entertainment and social media was then redirected to studying. In addition, participants expressed that reducing entertainment time also improved their time management, which they previously found challenging. Participants further expressed that they had to learn to plan weeks ahead, so that they could manage the theoretical and clinical demands. Planning a week ahead would include allocating times to study individual modules as per the drafted plan:

*“So, what I found that was really helpful is to take a day in a week, for example a Sunday, and just to plan out the next week. So, make sure that you have everything ready for the next week…”*

*“So, my strategy is to cope with that. I’ve been trying to eliminate my social life, like I’ve been trying to reduce the things that I do socially, like entertainment.”*

### Sub-category 2.5: social coping

Social coping is described as seeking support from the community or society to enhance ability to cope with a stressor [14]. Participants expressed that they had to form relations and associate with other students in order to cope with the transition from basic to higher education. They explained that the persons that they formed relationships with matured into becoming study partners and assisted them with interpretation services to patients. The participants stated that social coping was also used to try and understand the experiences of their peers, which they could relate to. They explained that they would normally sit in groups after class and talk about their experiences and how each is navigating the stressors associated with the transition from basic to higher education. In addition, participants explained that they would find humour about their experiences during those group sessions as a way to cope, and not necessarily to make a mockery of the situation:

*“… they placed us in hospital mixed ratio, like with when I work with my one friend, she can speak Setswana then the two of us will work together the whole time so that she can speak to the patient when the patient is Setswana, and I can speak to the patient when the patient is Afrikaans.”*

*“… we used to finish at around half past 11–12 and then we had our community health class starting at 3. So, that hour between our session… we would sit in our nursing group our like clique [showing quotation signs with hands], we would sit and just talk and we would basically make light of our situation.”*

### Sub-category 2.6: seeking help from mentors

Another coping strategy that participants used was seeking help from mentors. The identified mentors were senior undergraduate nursing students, supplementary instructors, and (to a certain extent) student leadership structures. Participants expressed that they coped by seeking advice from their seniors within the Bachelor of Nursing program. They explained that they would periodically ask their seniors how they coped when they were faced with similar adversities and would apply those coping strategies. Furthermore, participants expressed that having some guidance from supplementary instructors assisted them to cope with the academic demands. This is particularly important because in most cases supplementary instructors would be people in the same age group as the students, who they could therefore relate to better, and could maximize learning. Participants indicated that they also sought help from their lecturers and parents in order to cope with the transition from basic to higher education:

*“I will normally ask those that have experienced the first year, those that were once first years, how do you cope with this thing on the whole concept of nursing? How do you deal with your life, your personal life and your academic life?”*

*“What I wanted to say is some of the children, when they encounter situations where nurses are unfair or not treating them right, they do… I don’t know, here at the university they can… I don’t know how to say it in English. They can go give those names, like writing it, write a report and give their names to the faculty.”*

## Category 3: recommendations for coping with the transition from basic to higher education

The third category, recommendations for coping with the transition from basic to higher education, had seven sub-categories, which are elaborated on below.

### Sub-category 3.1: academic support

Participants suggested that academic support would come in handy in ensuring a smooth transition from basic to higher education. Within academic support the recommendation that they highlighted was the need to have additional study materials, such as recorded lessons and tutorial videos of the content being taught in class. In addition, participants recommended that the time spent in the lecture room should be reduced to allow them to prepare for other classes and even to study further what had been taught in class. Lastly, the participants recommended that assessment schedules should be shared well in advance so that they could have sufficient time to prepare for assessments amid their hectic schedules:

*“… if like they would upload maybe like tutorial videos on the content or study unit, that would be much better.”*

*“… they don’t take too much of our time, so that we can just use half of the day to study then prepare for the practical. Then when we get back from the practical, so that we can also prepare for the next class.”*

### Sub-category 3.2: informational support

Participants expressed that there is a need for a contextual orientation program for nursing students that is based on their needs. They explained that the orientation program offered at higher education, which focuses on showing students landmarks of the university, is not that helpful to nursing students. They expressed that having someone to explain the logistics of nursing to undergraduate first-year nursing students during the orientation period could be helpful. They explained that this would help undergraduate first-year nursing students to be prepared for first year of the Bachelor of Nursing program, because they would be aware of the expectations of the program:

*“Maybe if the coming first years should at least be orientated on what really happens in nursing. Unlike the orientation that took place, whereby we were shown around the school …”*

*“I think there should also be better procedures put in place in terms of the navigating your way around campus. It’s a bigger place than high school.”*

### Sub-category 3.3: psychological support

Participants expressed that there is a need for stress management sessions for undergraduate first-year nursing students, as these would greatly assist the students to debrief about the adversities they face during the transition from basic to higher education. Thus, would improve their ability to cope. They further suggested that the stress management sessions could be done in the form of group counselling, since they all experience similar adversities:

“… *think of a stress management because nursing can be stressful. So, teaching stress management techniques like meditating, it can be beneficial.”*

*“Then another thing that I think might be effective is group counselling sessions that’s specific to nursing and the health care.”*

### Sub-category 3.4: support from mentors

The participants expressed that they could cope better if lecturers or clinical preceptors would check on them from time to time in terms of how they are coping, especially with regard to their clinical experiences. They explained that such support should not only be regarding negative experiences, but also to celebrate milestones in clinical placement. Furthermore, participants suggested that clinical placement should be structured in such a way that it encompasses senior students. They indicated that, clinical preceptors have a large pool of students to supervise and might not always be around. Being placed with senior students gives them a fair amount of confidence, because they know they can always consult them since they are available:

“*I think making an effort to ensure that there is another senior on the ward with you, I think that will also help with you feeling secure about what you are doing.”*

“… *I know that there is a senior member that if anything happens, if there is a situation, because I know it’s not always necessary to call the preceptor because sometimes is just ‘Oh! I’ve seen my first patient die’, or ‘Oh! I just witnessed a terrible situation’ …”*

### Sub-category 3.5: external support

Participants recommended that lecture periods should be reduced to allow time for self-study and for preparation for commitments such as clinical placement. They also suggested that theory and clinical days should be separated to ease navigation between the two competing demands of the Bachelor of Nursing program. They explained that they would recommend that theory time be completely separated from clinical time, a system called a block system, where students attend classes for a certain period and later attend clinical placement for an agreed period. In addition, participants expressed the need for supportive messages on the institutional teaching and learning online platforms. They explained that lecturers should sometimes send out supportive messages using online learning platforms, instead of communications that remind them of their workload all the time.

*“The lecture should be reduced; the time should be reduced for us to go study and also the practical.”*

*“… going to class today, tomorrow we’re going for practical, it’s really draining. Like if maybe they can do … this week it’s classes, then next week its practical, just like that, because it’s really difficult…”*

### Sub-category 3.6: opportunities to improve communication

In terms of opportunities to improve communication, participants recommended that undergraduate first-year nursing students should be afforded a compulsory vernacular subject on the language that is common in the community where the university is located. They explained that such a vernacular subject should be an addition to their mother tongue and English, which can enable them to communicate with patients and fellow students with ease. They also suggested the need for social activities, which will pave the way for socialization among students from different cultural and geographic backgrounds. They also recommended that there should be an anonymous channel for students to communication their concerns to lecturers or other students; this would be helpful because some students find it difficult to express themselves:

*“I think it will really benefit us to have maybe one semester or even a first year to have a different language that you can learn…”*

*“So it is much better for you to actually write something that to send in your request anonymously, to say ‘Ma’am, this is what I request’”.*

### Sub-category 3.7: opportunities to release stress

Participants recommended that apart from teaching and learning activities, there should be opportunities to release stress, as a way to cope with the transition from basic to higher education. Opportunities suggested by the participants were a sports day for nursing students, being afforded an opportunity to enjoy recess, and periodic team-building events where they can meet with nursing students from other universities. They expressed that these opportunities would allow them to socialize and strengthen their coping mechanisms:

*“I don’t know if it’s late or what, like maybe like a trip somewhere, like for a weekend. Get to meet like professional nurses. Like so that we can meet with other first-year nurses from maybe other universities, so that we meet and talk, some motivation.”*

*“There should be a sports day for nursing students where they go and play basketball and netball, football.”*

## Discussion

The main aim of this study was to explore and describe the coping strategies used by undergraduate first-year nursing students during their transition from basic to higher education at a South African university. Data was sourced from undergraduate first- year nursing students during the second semester of their training. The major findings of this study were their experiences of the transition from basic to higher education, strategies for coping with the transition, and recommendations for coping with the transition from basic to higher education. Although the study was conducted during the denouement of COVID-19, no evidence suggested that this influenced the findings, which seemed to be generic in nature rather than related to the impact of the COVID-19 pandemic.

## Experiences of the transition from basic to higher education

Participants expressed that their experience of transition from basic to higher education means transition to independency and to demanding schedule. These findings are corroborated by Hughes et al., [8] who reported that transition to higher education for undergraduate first year nursing students implies learning through demanding schedules and transitioning to self. Participants further expressed that, during the transition, they had to learn to adapt to academic and clinical demands and be able to balance between the two. Likewise, Hughes et al. [8]. found that undergraduate first year nursing students often find it very difficult to balance between theory and clinical demands. Thus, making first year of the Bachelor of Nursing degree challenging. As a result, majority are likely not to cope with such transition. Adapting to social demands was also flagged as an experience which participants had to endure during their transition from basic to higher education. They explained that transition from basic to higher education was coupled with language and culture shock. A study conducted by Stenberg et al. [15] reported similar findings where they found out that culture and language are one of the barriers to smooth transition from basic to higher education. Therefore, based on the findings of the study, the authors argue that transition from basic to higher education is a challenging exercise which requires viable coping strategies. The author further argues that the challenge in transition from basic to higher education might be accounted for by the consequences of COVID-19.

### Coping with transition from basic to higher education

The participants showed the need for viable coping strategies owing to the challenges they face during the transition from basic to higher education. They argued that the orientation period that is meant to smooth their transition from basic to higher education is not content-specific to the needs of undergraduate first-year nursing students. This was in contradiction with Rossato et al., [2]whose findings indicated that welcoming activities help students to adapt to the nursing course in their first year. The findings revealed six strategies that the participants use to cope with the transition from basic to higher education: adaptive coping strategies, appraisal-focused coping strategies, emotion-focused coping strategies, problem-focused coping strategies, social coping strategies, and seeking help from mentors. In their study, Andrew et al. [16] stressed that high-quality mentoring and breaks in between theory and clinical placement improves students’ ability to cope with the transition to higher education. Furthermore, a study conducted by Monisha et al. [17] reported that good psychological and behavioural coping strategies are essential in improving the transition from basic to higher education, thus confirming the findings of the current study. However, a study conducted by Bodys-Cupak et al. [4] found that in addition to the coping strategies identified in this study, some students use destructive coping strategies such as the use of psychoactive substances. In this study issues related to psychoactive substances were not mentioned among the coping strategies.

The findings support a call for contextual structured support for undergraduate first- year nursing students that will help to smooth their transition from basic to higher education. It is argued that the coping strategies mentioned here should be incorporated into the orientation program to improve the resilience of undergraduate first-year nursing students during their transition from basic to higher education.

### Recommendations for coping with the transition from basic to higher education

The findings of this study showed that there is a need to strengthen academic support and informational support by means of contextual orientation programs, psychological support, and support from mentors, to improve undergraduate nursing students’ transition from basic to higher education. In addition to the aforementioned recommendations, participants indicated that there is also a need for external support by alleviating the nursing program so that they can also be integrated into the university activities. They explained that the current structure of the program is very busy and does not allow them adequate time to plan their academic activities, let alone be able to socialize. Consistent with the recommendations in this study, Andrew et al. [16] found that undergraduate first-year nursing students who have additional mentorship and informational support in online resources transition better to higher education from basic education.

## Conclusion

The transition from basic to higher education is challenging for undergraduate first- year nursing students, and a significant number of these students do not cope with it well. Hence the transition from basic to higher education requires effective coping strategies. The findings of this study highlighted the experiences of undergraduate first-year nursing students regarding the transition from basic to higher education, their coping strategies, and recommendations for coping with this transition. The findings affirm that there is a need to standardize and contextualize the support for undergraduate first-year nursing students during their transition from basic to higher education.

### Limitations

The study was conducted using an online platform, which made it somewhat difficult to record field notes, which might have influenced the findings. There was gender inequality among participants, most of whom were female, which might have influenced the results. Data was collected using focus group discussions, which might have influenced the results, especially if there were participants who were not comfortable speaking in a group.

## Data Availability

The data set used for compiling this manuscript is available from the corresponding upon a reasonable request.
